# Supplementary material for: Deformation of Gels with Spherical Auxetic Inclusions
Source: Gels. 2022 Oct 29;8(11):698. doi: 10.3390/gels8110698 (PMC9689618; doi:10.3390/gels8110698)
Supplement: Supplementary file 1 [file gels-08-00698-s001.zip › gels-1970954-supplementary/Zidek_et_al_Supplementary1.pdf]

Article: Deformation of the materials with spherical auxetic inclusions

Supplementary 1: Tutorial of simulations

Jan Zidek[a**], Petr Polacek[a], Josef Jancar[a*]

[a]*CEITEC, Brno University of Technology, Purkynova 123, Brno, Czech Republic*

**questions and comments to the tutorial, please send to email: jan.zidek@ceitec.vutbr.cz

*corresponding author (article): jancar@fch.vutbr.cz

1. Introduction

The document presents detailed tutorial for programming of the models, running the simulation and data evaluation. The user needs three tools: MATLAB, GIBBON-code library, and FEBIO software. For the purpose of the paper, all the programs were installed on WINDOWS 10 platform. All software packages are available versions for LINUX, UNIX.

The basic environment for control simulation was MATLAB software. Actual paper was performed with MATLAB version 9.4.0.813654 (R2018a).

The control of simulation was designed by the Gibbon scripts. Actual paper was performed with version downloaded on 25.3.2021. The Gibbon scripts run in MATLAB environment. GIBBON is freely distributed under GNU license.

The simulation tool is FEBIO software. Simulation in actual article were provided by version FEBIO 3.3.1. The FEBIO is distributed under University of Utah license.

The GIBBON code includes functions for definition of the initial structure, settings of simulation and processing of resulting data. It runs the FEBIO software.

2. Before the first simulation

1. Install the **FEBIO** software first
2. Install the **GibbonCode** (set the path to executable file for FEBIO, febio3.exe)
3. The GibbonCode contains the directory: **/docs**
4. Open the directory docs in **MATLAB**

Each simulation is performed by script in MATLAB. The functions and orders from following chapters must be added to the file.

3. Detailed model of porous hexagonal honeycomb structure

This section describes building of the model structure composed from conventional and re-entrant cells. The auxetic behavior was included by specific shape of structure. The contours of the model are determined by the coordinates of vertices. The object borders are defined by parametric definition of volume elements.

2.1 Step1: Parametric definition of the 2D-honeycomb mesh

First step is a definition of the size of the system. Size is the number of honeycomb cells (nx, ny) in one coordinate system. The model of 2D-hexagonal honeycomb network was calculated (Figure S1).

Function: honeyCombMesh, from Gibbon-library

Input Data: Dimensions of lattice (nx, ny) **Output Data:**

V-Vertices of hexagonal network, **V**=$\{V_1, V_2,\ldots,V_n\}$; where $V_i=(x_V, y_V, 0)$

F- grouping of hexagonal vertices to the hexagonal elements **F**=$\{F_1, F_2,\ldots,F_e\}$; where $F_1\ldots$ is a set of six indices: $F_j=\{i_1,i_2,i_3,i_4,i_5,i_6\}$

Actual value: honeyCombMesh(10, 10)

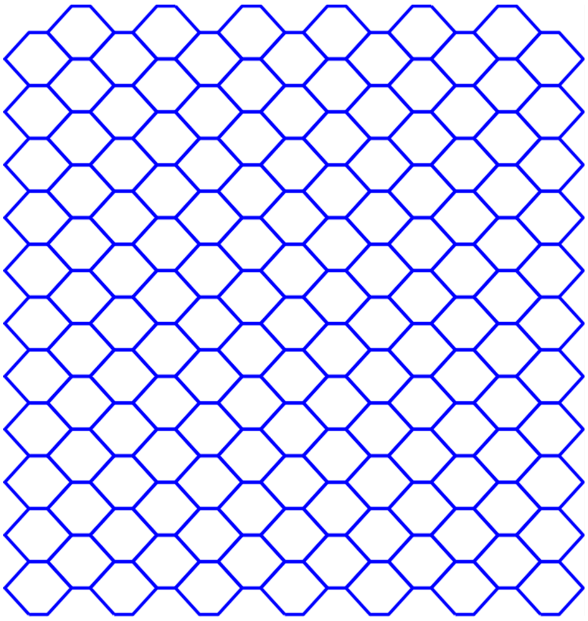

Figure S1.1: 2D-xy-honeycomb lattice {honeyCombMesh(10, 10)} as a basic building block of our auxetic structure

2.2 Step2: 3D-honeycomb mesh from 2D matrices

The 3D pattern of Honeycomb cells composed from (2D meshes) wireframe model.

Function: wireframe model; our in-house script

Input Data: Hexagonal lattice

Output Data: V-Vertices of wireframe model, $\mathbf{V}=\{V_1, V_2,\ldots,V_n\}$; $V_i=(x_V, y_V, z_V)$

P- Pairing the (linking) between two vertices with indices *i, j*. $\mathbf{P}=\{P_1, P_2,\ldots,P_e\}$; $P_k=\{V_i,V_j\}$

The wireframe model was programmed by our in-house script. It was constructed from a set of honeycomb lattices. One 2D-xz-honeycomb lattice was copied to the parallel and aligned honeycomb arrays (blue-Figure S1.2 left). The set was copied to the set was copied to 2D-yzlattice (red-Figure S1.2 left). The existing connections of vertices in honeycombs were cancelled and replaced by new connections (Figure S1.2 right). The results are the array of 3D cells where the projection into xz or yz plane result in a 2D honeycomb array. A result was parameterized system of pair wire connections and coordinates of vertices.

That model has hexagonal pattern in projection to xz and yz plane.

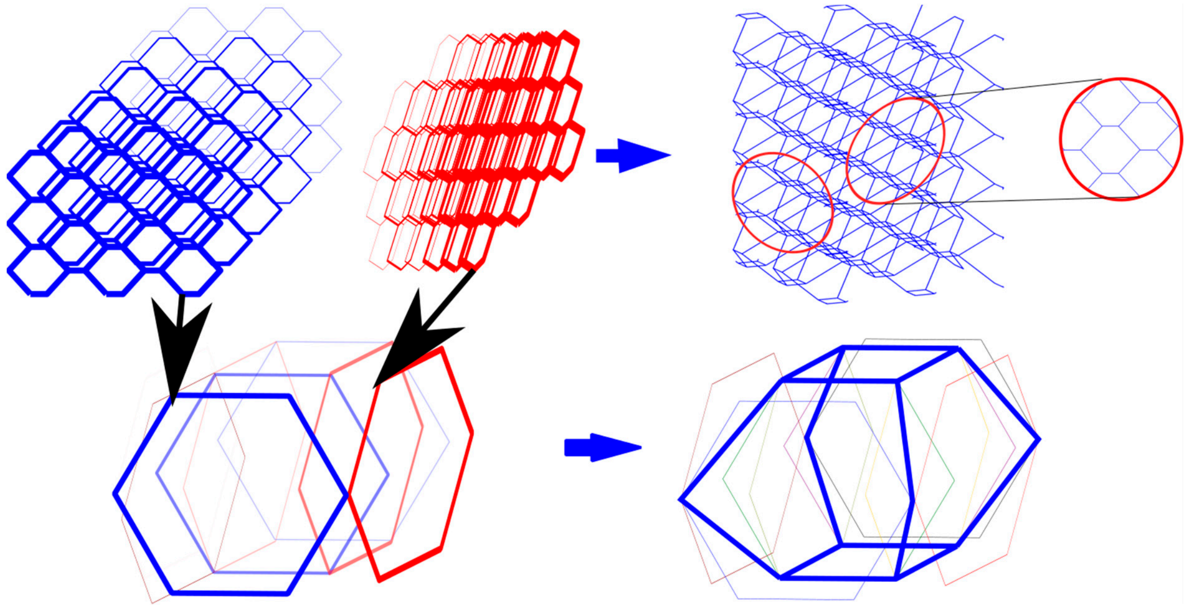

Figure S1.2: The construction of 3D-honeycomb wireframe model; the model is designed from aligned layers of honeycombs; xz and yz projection of the model is honeycomb.

2.3 Step3: Transformation of 3D-honeycomb to re-entrant 3D honeycomb

Function: Transformation to auxetic; our in-house script

Input Data: Wireframe model

Output Data: $\mathbf{V_{new}}$-new coordinates of vertices

P-pairing of vertices is identical like in previous function

Selected conventional cells were transformed to the auxetic cells. The transformation was performed by a horizontal shift of the vertices (Figure S1.3). The transformation step was applied to selected cells. The other cells were left conventional. The cells inside the spherical inclusion were transformed to re-entrant.

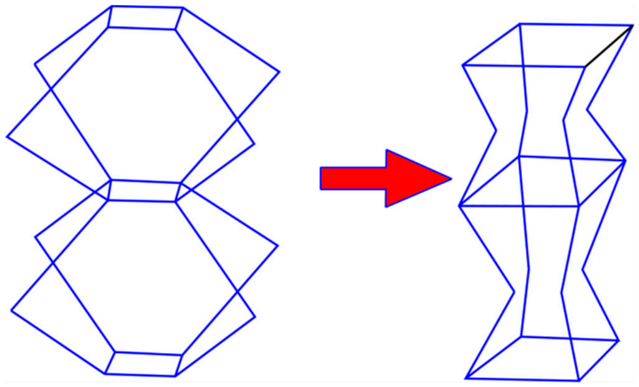

Figure S1.3: The transformation of the wireframe model cells from Figure S2 to the auxetic version.

2.4 Step4: Transformation to volume elements

The 3D FEM model is based on 3D elements. The wireframe model has zero volume. It was transformed to the volume elements. The horizontal lines were transformed to blocks (Figure S1.4).

Function: Transformation to volume elements

Input: wireframe model, thickness, width of lamellas

Output: **V**-vertices of the model, **E**-grouping to the elements; **F**- facets; **C**-material identifier; **Fb**-boundary facets; **Cb**-identifier of surface

Transformation of wires to volume elements was performed by our script. The wire connecting two lines was transformed to the hexahedral (cubic) elements.

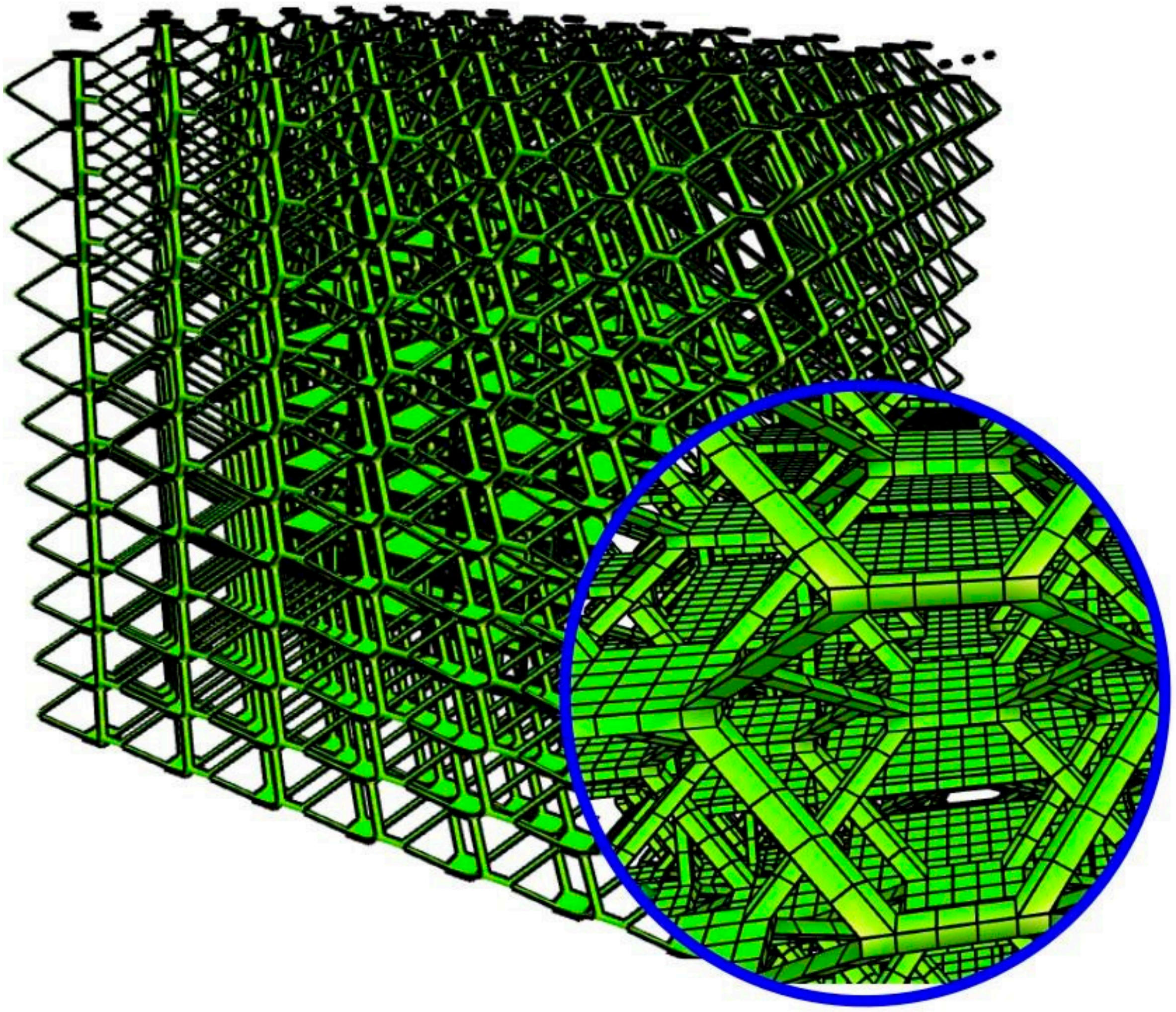

Figure S1.4: 3D volume model of the honeycomb materials. The connection lines are the beams in the material; inset: detailed view to single model cell of the 3D material.

Now, the model 1 structure is done. Its deformation is presented in Figure 1a of the main text.

4 Coarse model (solid spherical inclusions in continuous matrix)

This section contains definition of volume of the next macroscopic model. The auxetic structure is included by settings of negative Poisson's ratio in the spherical inclusions. The output structures are vertices and their grouping to the volume elements.

The basic input is a set of coordinates and radii of all spheres and size of box.

3.1 Input data

It is considered that the radius of sphere is 1 (length unit). There is necessary to know the coordinates of sphere centers and size of box. The coordinates of centers are ordered to the face centered cubic (FCC) lattice. However, the spheres can be ordered randomly. The next parameter is a size of box.

The next parameter is a resolution (number of triangles related to plane area).

Table S1.I Input properties of the model.

**Property**	**Name**
s_b	Size of box
r_b	Resolution of box
**CS** = {$\mathbf{CS_1}$, $\mathbf{CS_2}$,…, $\mathbf{CS_n}$}; CS_i ={CS_x,CS_y,CS_z}	Centers coordinates
d_s	Degree of resolution of spheres

3.2 Building of box

Box is a cubic object, which is defined by a triangulated surface of box

Input: size of box (s_b), degree of resolution of box (r_b)–elements/box side

Function 1: quadBox, included in Gibbon-code

Output 1= Input 2: V-vertices of surface square elements; F- indices of vertices

Function 2: quad2tri, included in Gibbon-code

Output 2: V-vertices of surface triangular elements; F- indices of vertices (Figure S5a)

3.3 Building of inclusion

Inclusion is a spherical object defined by a triangulated surface of the sphere

Function: geosphere, included in Gibbon-code

Input: radius of sphere, degree of resolution

Output: triangular elements of the sphere surface (Figure S5a).

3.4 Completing of the model

Generation of 175 spherical objects and shifting to the coordinates inside box. The surface of spheres cannot intersect with the surface of box. Neither two particles cannot intersect.

The spheres were ordered to Face Centered Cubic (FCC) lattice, however, they can be ordered to any distribution of spheres. Thereof FCC and Hexagonal Close Packing (HCP) are the most uniform and enable one to fill up to 74 volume%.

The alternative ordering are cubic lattice which enables one to fill the space with 52 volume% of inclusions. Next there is possible to set random distribution of spheres for example by AGLOMER software.

The FCC was selected because there are the most uniform distribution of nearest distances between spherical inclusions.

3.5 Volume elements

The surface contours were transformed to volume elements.

Function: runTetGen, in Gibbon-code

Inputs: Structured surfaces of cube and spheres.

Output: The model creates the 3D-tetragonal lattice, the results are the elements assigned to the appropriate phase (blue matrix-phase 1 and red particle-phase 2 Figure S5b.).

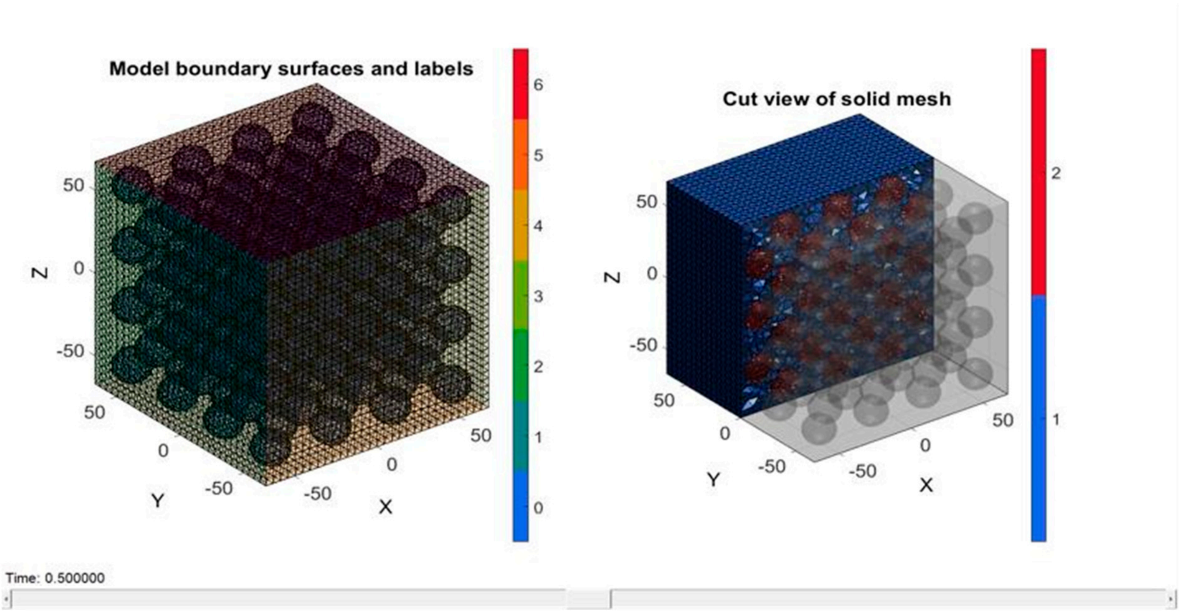

Figure S1.5: a. components of the model box and 172 spheres; b. cut inside the mesh.

The parameters of the lattice are V-vertices of the model, E-grouping to the elements; F- facets; C-material identifier; Fb- boundary facets; Cb-identifier of surface The structure from Figure 2 of main text is done.

The next step is transformation of geometry to the real behavior of solid objects.

The GIBBON-code

1. edits the input file for FEBIO software;
2. controls the running of FEBIO;
3. reads output FEBIO files.

5 Editing the input files

The input file is coded in MATLAB structure array (name: febio_spec). It is suitable analytical tool, how to define material from different aspects: structure, material, mode of deformation.

4.1 Introduction of model geometry

Geometry of the model was included as a 3D mesh of the materials

List of all vertices in the mesh: **febio_spec.Mesh.Nodes**

List of all elements in the mesh: **febio_spec.Mesh.Elements**

List of surface elements in the mesh: **febio_spec.Mesh.Surfaces**

Definition of node Set: selection of vertices **febio_spec.Mesh.NodeSet**

The operator can select the groups of vertices for certain defined actions. One can apply displacement, force, fix the nodes.

4.2 Assignment of materials properties

Definition of material: **febio_spec.Material.material**

Operator can define material characteristics, for Example neo-Hookean material with modulus 2.5 MPa and Poisson's ratio -0.9

febio_spec.Material.material{2}.ATTR.name=materialName2;

febio_spec.Material.material{2}.ATTR.type='neo-Hookean';

```
febio_spec.Material.material{2}.ATTR.id=2;
febio_spec.Material.material{2}.E=2.5; febio_spec.Material.material{2}.v=-0.9;
```

Domain in structure was defined in Mesh section by selection of elements. All elements inside spherical inclusions were assigned as the Domain 2: **febio_spec.Mesh.Elements{2}.ATTR.name=partName2;** Assignment of materials to the structural domains:

For example, the Material2 (auxetic material) was assigned to the spheres.

```
febio_spec.MeshDomains.SolidDomain{2}.ATTR.name=partName2;
febio_spec.MeshDomains.SolidDomain{2}.ATTR.mat=materialName2;
```

4.3 Boundary conditions

The boundary conditions (bc) are intended to control the deformation. At the same time, the bc are important for the solution of differential equation in the modeling software engine, in order to find the stable solution of differential equations in the model.

There were added two types of boundary condition. It is fixing the lower plane (Figure S1.6, lower plane) and prescribed displacement of upper plane.

Example 1: fixing of lower plane in x-direction,

```
febio_spec.Boundary.bc{1}.ATTR.type='fix';
febio_spec.Boundary.bc{1}.ATTR.node_set=nodeSetName3;
febio_spec.Boundary.bc{1}.dofs='x';
```

Example 2: Prescribed displacement of upper plane in x-direction,

```
febio_spec.Boundary.bc{6}.ATTR.type='prescribe';
febio_spec.Boundary.bc{6}.ATTR.node_set=nodeSetName4;
febio_spec.Boundary.bc{6}.dof='z';
febio_spec.Boundary.bc{6}.scale.ATTR.lc=1;
```

febio_spec.Boundary.bc{6}.scale.VAL=displacementMagnitude;

febio_spec.Boundary.bc{6}.relative=0;

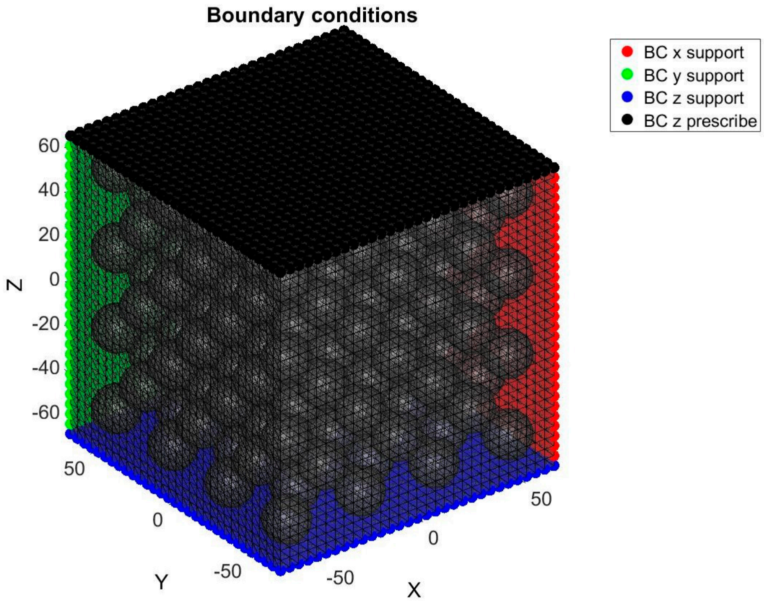

Figure S1.6: Definition of boundary conditions. Black (prescribe z) defined displacement of vertices; blue z-support fixed coordinates.

4.4 Control of deformation

The deformation is defined by range of deformation, type of deformation and in the case of dynamic simulations also the time of simulation. The time of simulation is defined by a struct: **febio_spec.Control.analysis='STATIC'; febio_spec.Control.time_steps=numTimeSteps; febio_spec.Control.step_size=1/numTimeSteps;**

Magnitude of displacement was defined in the boundary conditions: **febio_spec.Boundary.bc{6}.scale.VAL=displacementMagnitude;**

Coupling the time and magnitude (variable VAL) means that after one block of time (time_steps*step_size) the sample must be deformed by displacementMagnitude. The simulations of our materials in this article are not dynamic and thus the time scale does not play a role.

febio_spec.LoadData.load_controller{1}.ATTR.id=1;

febio_spec.LoadData.load_controller{1}.ATTR.type='loadcurve';

febio_spec.LoadData.load_controller{1}.interpolate='LINEAR';

febio_spec.LoadData.load_controller{1}.points.point.VAL=[0 0; 1 1];

4.5 Output data

The febio_spec object can direct, which data desired to export. It is for the saving of disk space. For example displacement:

febio_spec.Output.logfile.ATTR.file=febioLogFileName;

febio_spec.Output.logfile.node_data{1}.ATTR.file=febioLogFileName_disp;

febio_spec.Output.logfile.node_data{1}.ATTR.data='ux;uy;uz';

febio_spec.Output.logfile.node_data{1}.ATTR.delim=',';

4.6 Other parameters

The other parameters are choice of version and module

febio_spec.ATTR.version='3.0'; febio_spec.Module.ATTR.type='solid';

6 Control of running.

The data produced in *Sections 2-5* van be saved to the xml file ('*.feb') and running the feb file from the FEBIO software.

Another alternative is to run it directly from the gibbon script

Function: runMonitorFEBio

The parameters are: filenames for input and log file

febioAnalysis.run_filename=febioFebFileName;

febioAnalysis.run_logname=febioLogFileName;

Switch whether the iteration outputs will be presented on the command line: **febioAnalysis.disp_on=1;**

```
febioAnalysis.runMode='external';%'internal';
```

The anti-looping condition:

```
febioAnalysis.maxLogCheckTime=1000; %Max log file checking time
```

6 Evaluation of output data:

The model produces output file is actual stresses, forces, and displacements of each vertices. It is meaningful to visualize or analyze selected data.

6.1. Movies of displacements or stresses in materials 6.2. Stress-strain curve and pressure-strain curve (Figure S1.7)

The next output was stress strain function and pressure-strain function

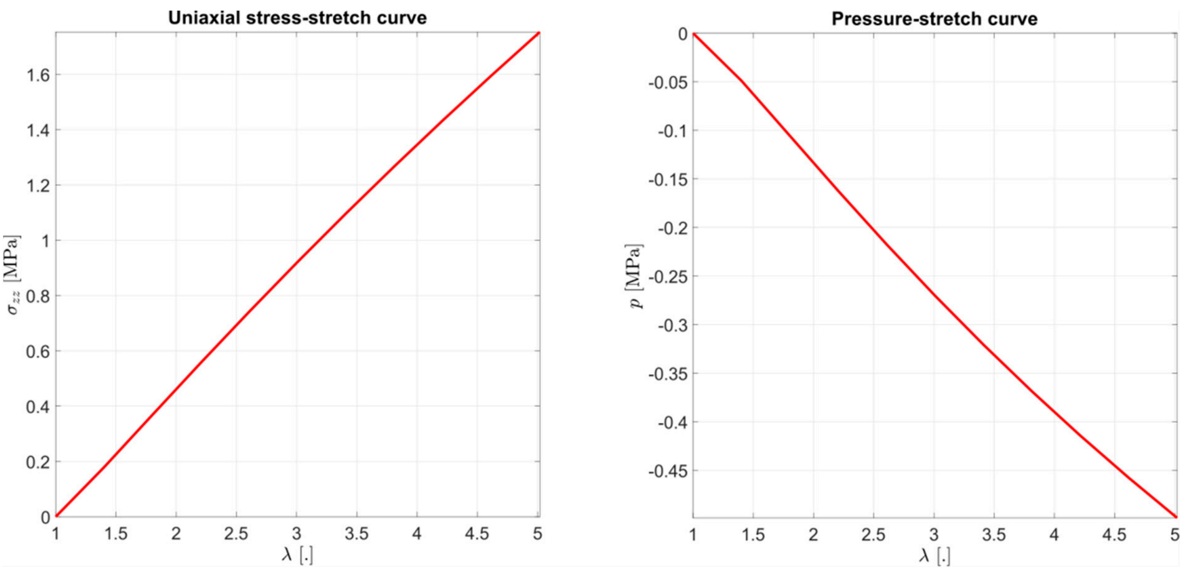

Figure S1.7: Stress-strain curve and pressure-strain function

6.3. Voigt array of stress

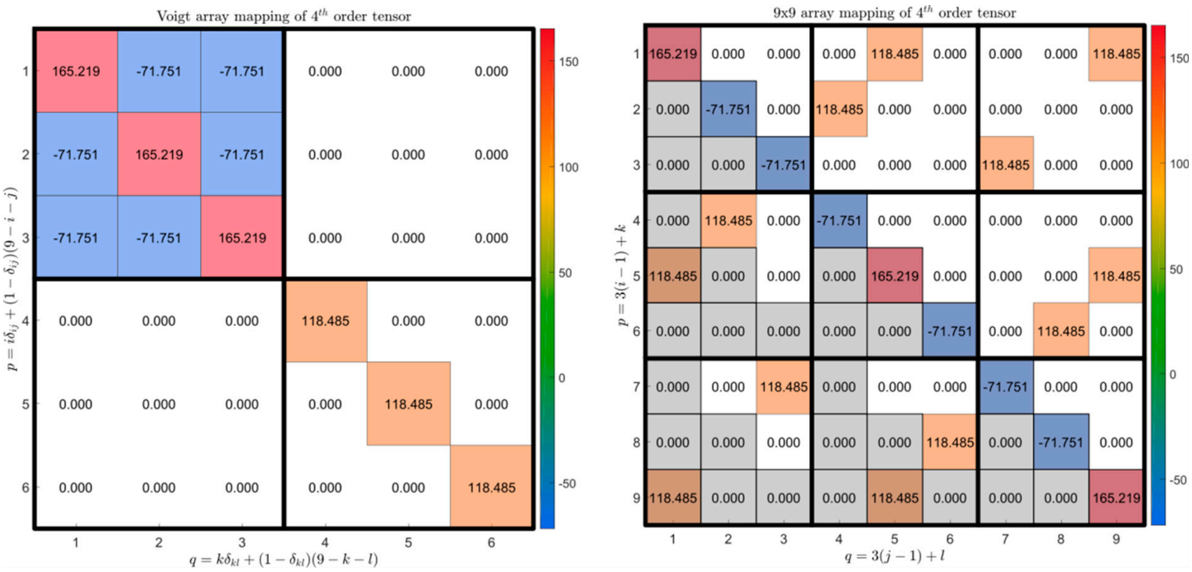

Figure S1.8: Voigt array matrix of the material.

6.4. Value of von Mises (Effective) stress of each element.

The von Mises stress is not basic mechanical property and it is not standard output from FEBIO/GIBBON script. There is needed to read stresses in all principal axes and then to calculate the von Mises stress from the Equation 21 in the main text. The result is a von Mises stress for each elements and coordinates of the center of element.

6.5. Visualization by FEBIO postview

The FEBIO postview proposes a visualization of all properties in different styles (slice plot, vector plot).
