# Supplementary material for: Deformation of Gels with Spherical Auxetic Inclusions
Source: Gels. 2022 Oct 29;8(11):698. doi: 10.3390/gels8110698 (PMC9689618; doi:10.3390/gels8110698)
Supplement: Supplementary file 1 [file gels-08-00698-s001.zip › gels-1970954-supplementary/Zidek_et_al_Supplementary2.pdf]

Article: Deformation of the materials with spherical auxetic inclusions

Supplementary 2: Direct outputs from simulations

Jan Zidek[a**], Petr Polacek, Josef Jancar[a*]

[a]*CEITEC, Brno University of Technology, Purkynova 123, Brno, Czech Republic*

**questions and comments to the results, please send to email: jan.zidek@ceitec.vutbr.cz

*corresponding author (article): jancar@fch.vutbr.cz

Introduction

The main article contains a selected images for illustration of the principles of deformation in the auxetic and composite material. The presentation of all data in the main document can reduce its clarity.

Our aim is to provide a complete set of data for all volume fractions: 5%, 10%, 20%, and 30%. The reader can find the data in separate files one pdf file for each Figures 1-6 from main text.

1. Figure 1 in the main text

Figure 1 in the main text shows highlighted traces of load transmission. This supplementary shows the outputs without graphical modifications

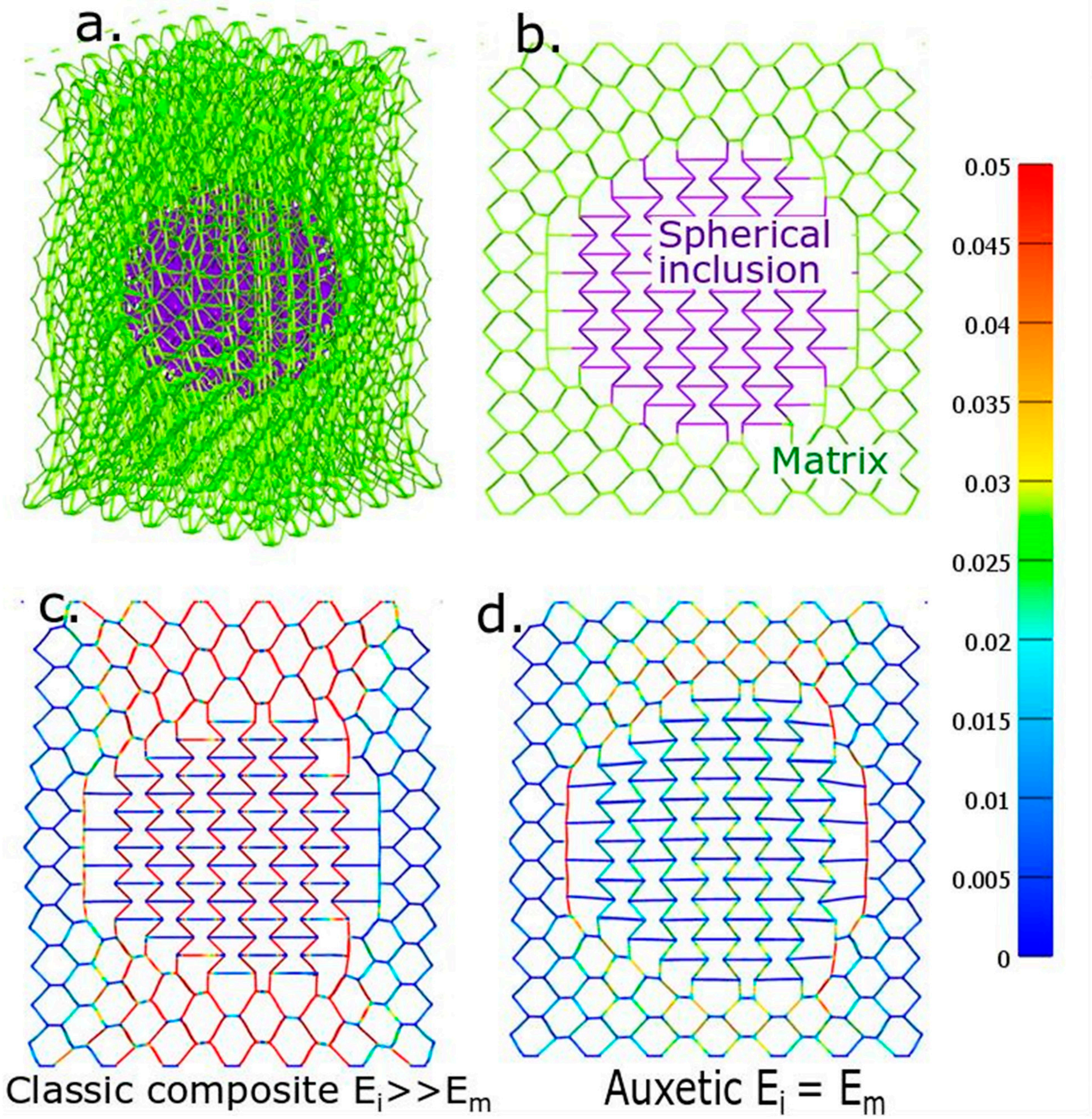

Figure 2 from main text

Figure 2 in the main text shows the cross-section of two samples with 30 volume % of particles: composite – rigid particles in deformable matrix, auxetic – auxetic particle in conventional matrix. The **supplementary shows** the stress distribution for all samples.

The snapshots for volume fractions were calculated (5%, 10%, and 20%). All the snapshots were selected with average stress 0.825 MPa. The neat matrix without filler has homogenous color. Entire cross-section in the sample has green color. There are only green domains of middle stress. Following figure shows the color scale-bar for stress (in MPa).

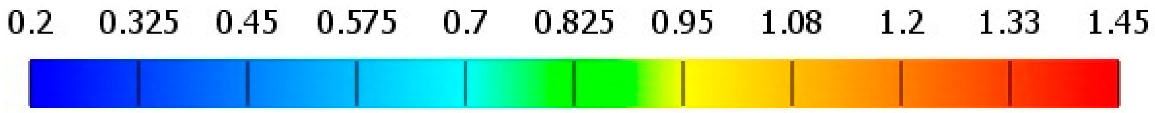

The same snapshots of samples filled with particles: solid particles, auxetic particles. Average stress in the sample is 0.825 MPa. The figures show distributions of strass in the cross-section of particles.

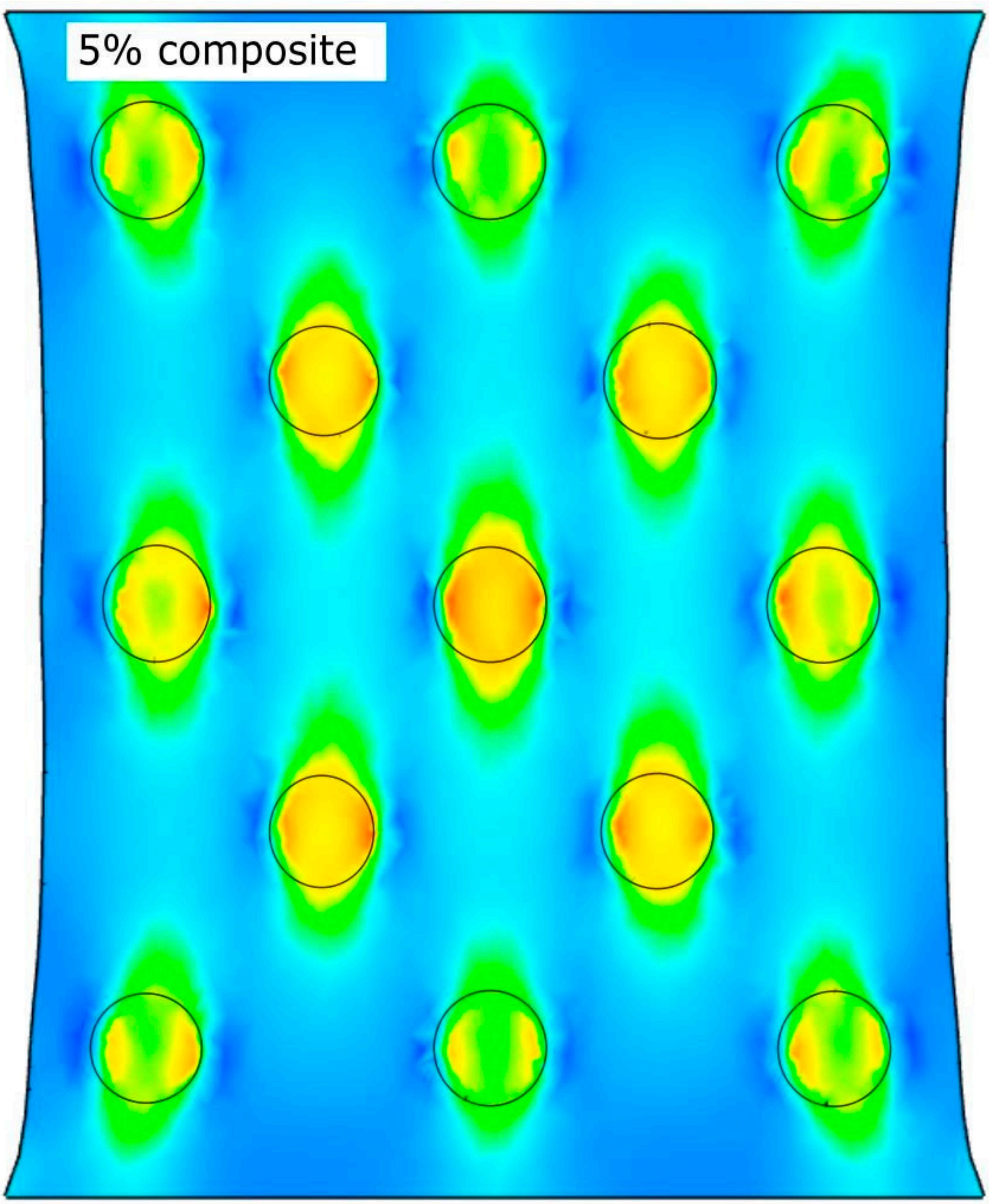
5% composite

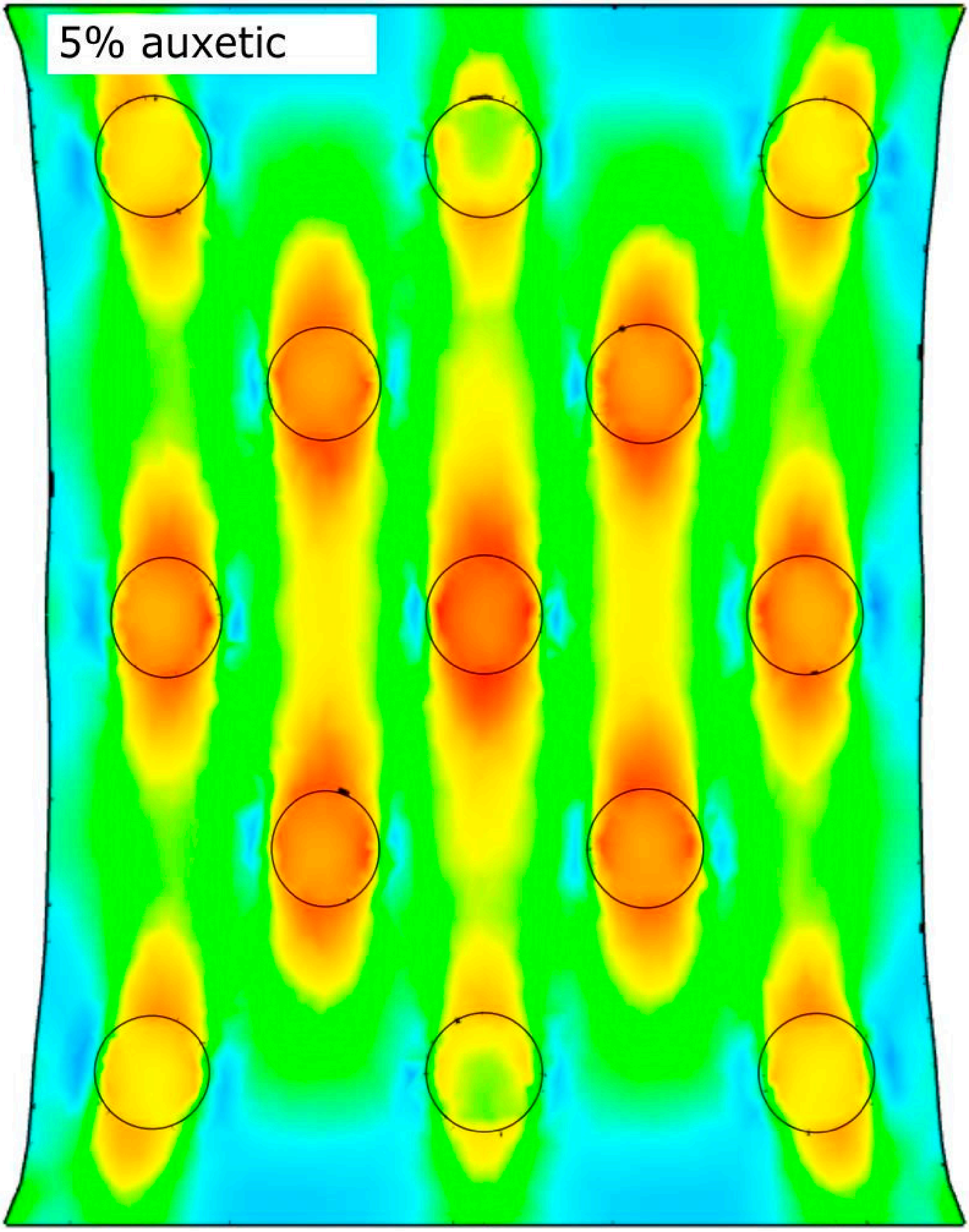
5% auxetic

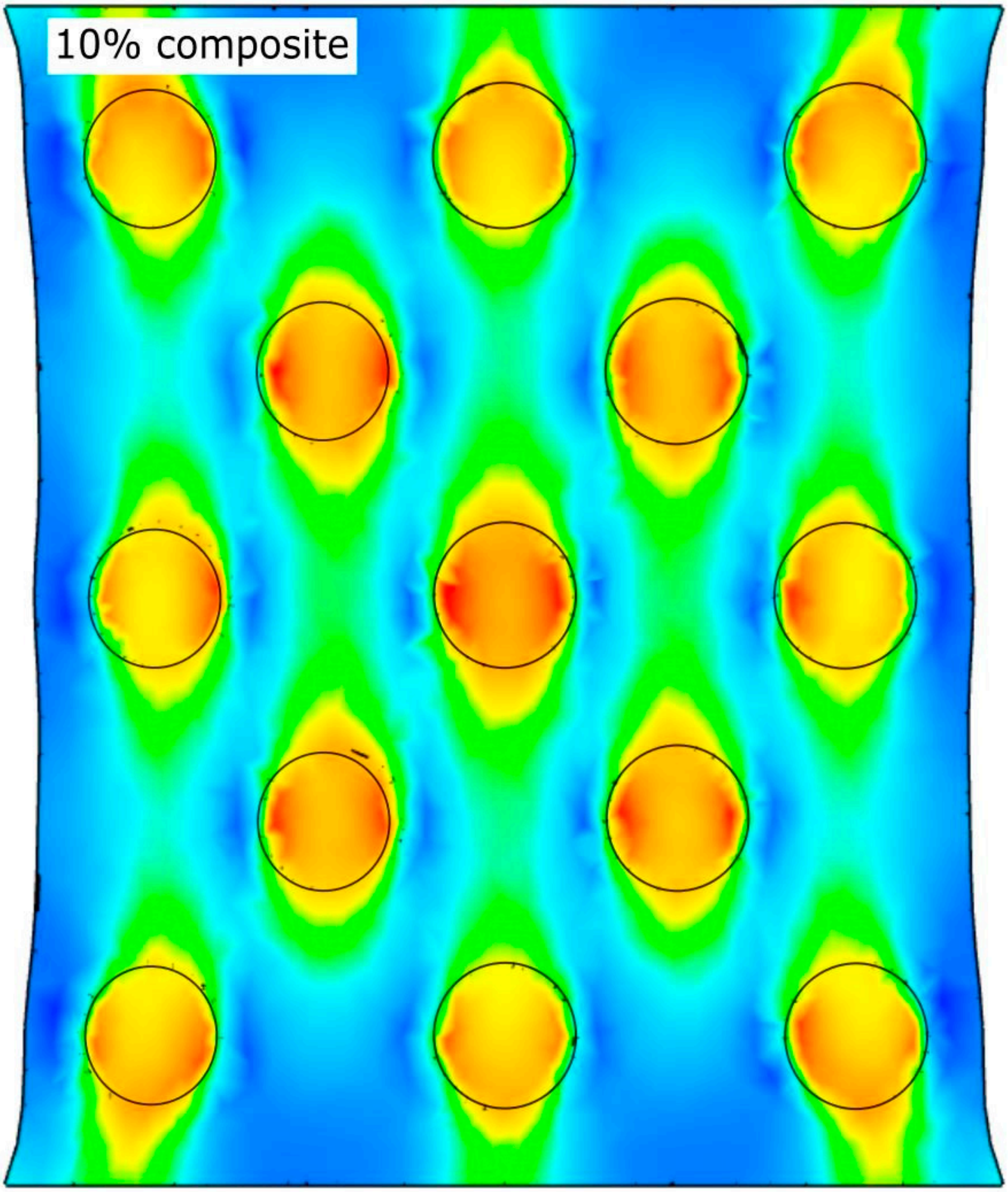
10% composite

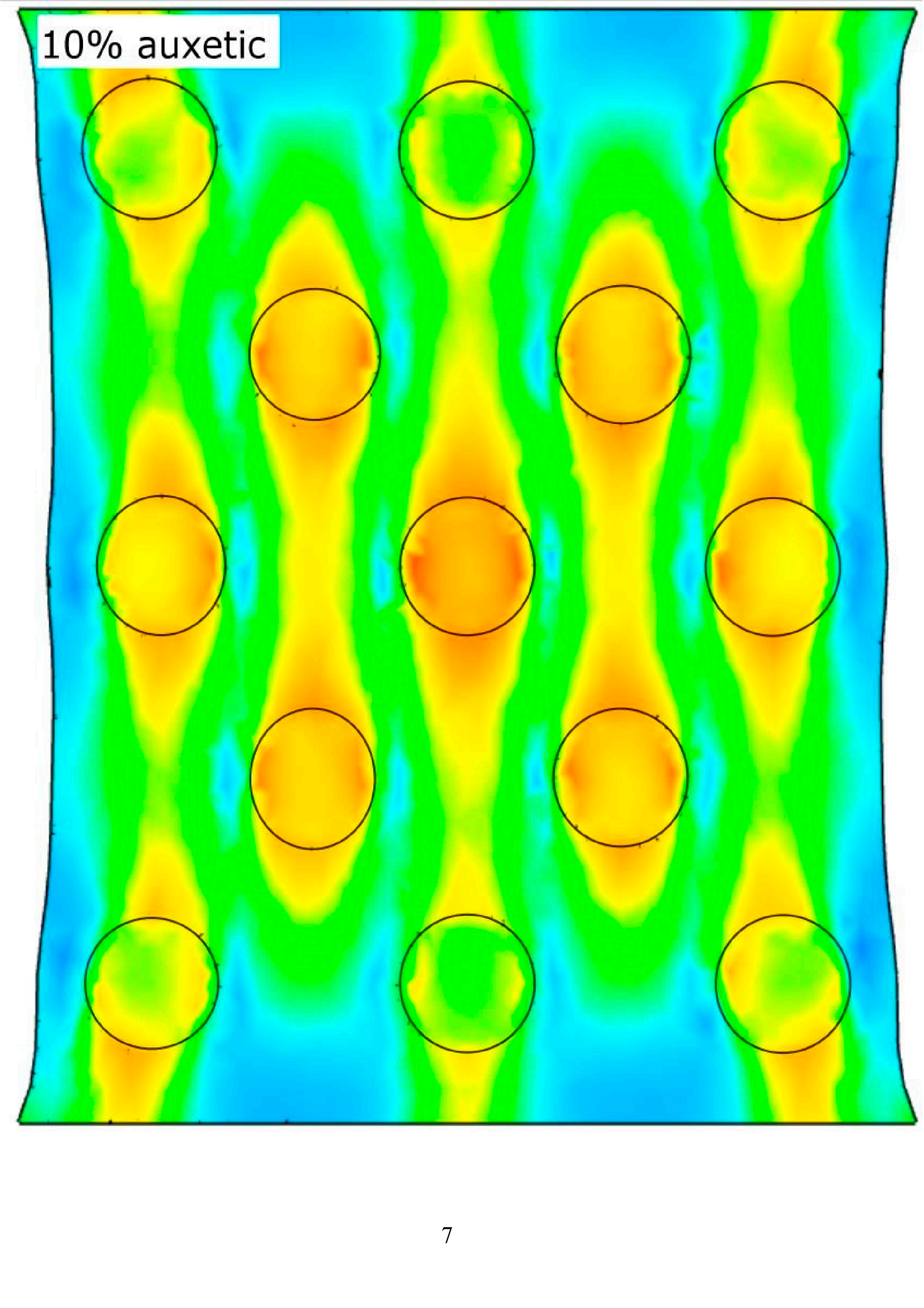
10% auxetic

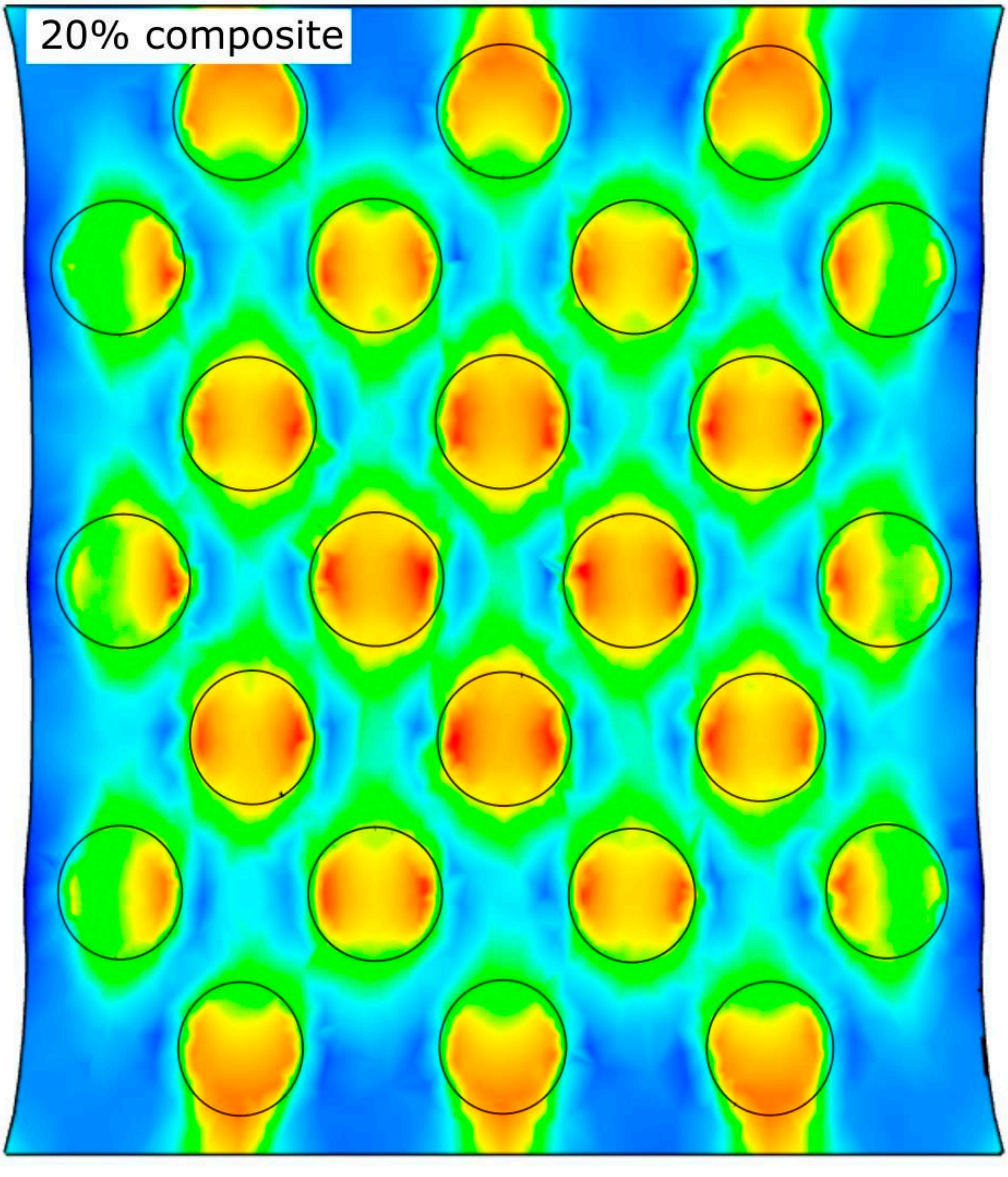
20% composite

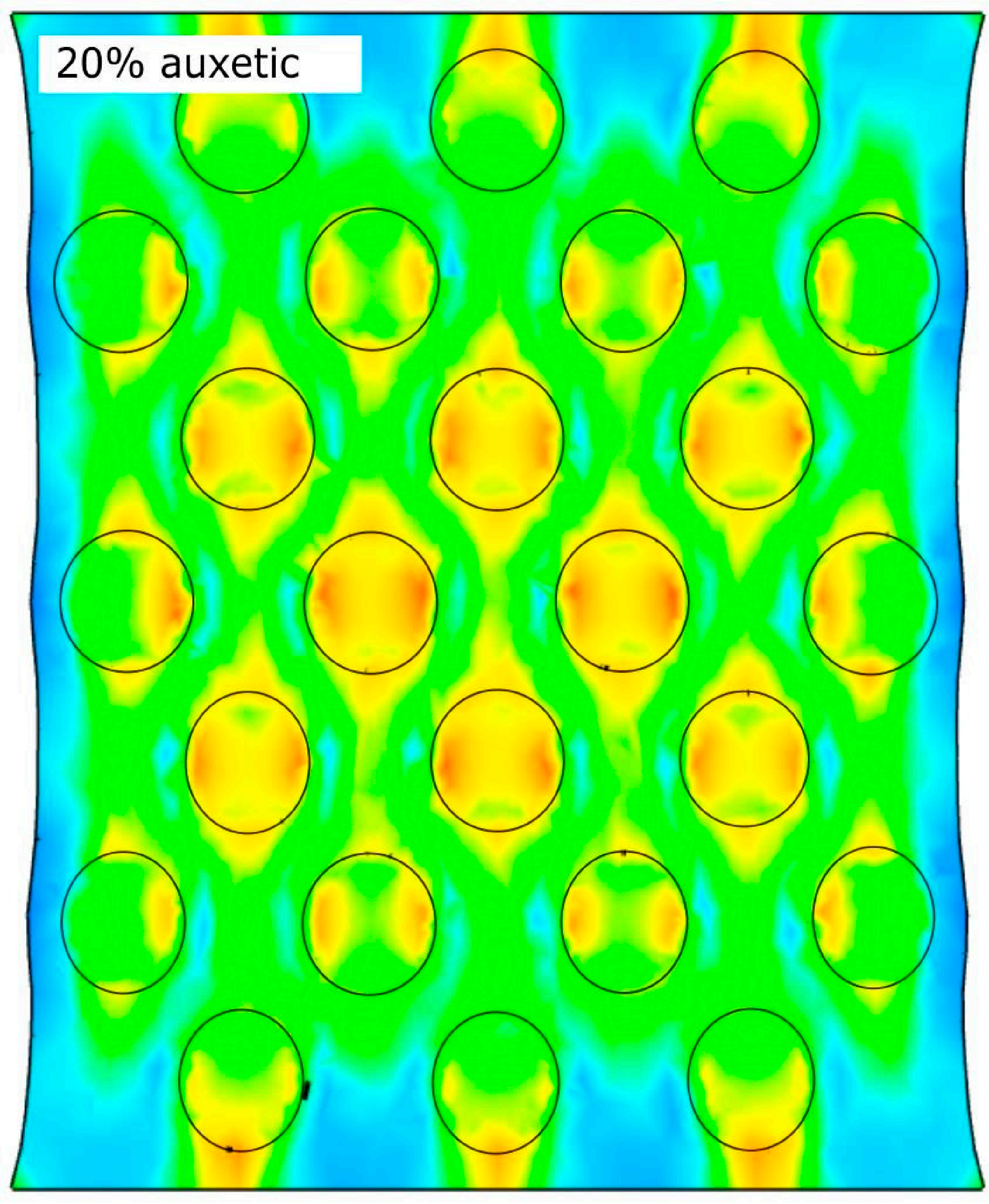

Stress distribution in the sample. Circles-rigid or auxetic particles.

Figure 3: Results from Mori-Tanaka model

The deformation behavior was calculated by a Mori-Tanaka model.

Main document shows the Young modulus from Mori Tanaka model in a range 0-40% of volume

The supplementary shows a value of Young modulus and Poisson ratio for all models from 0% to 40% of heterogeneities.

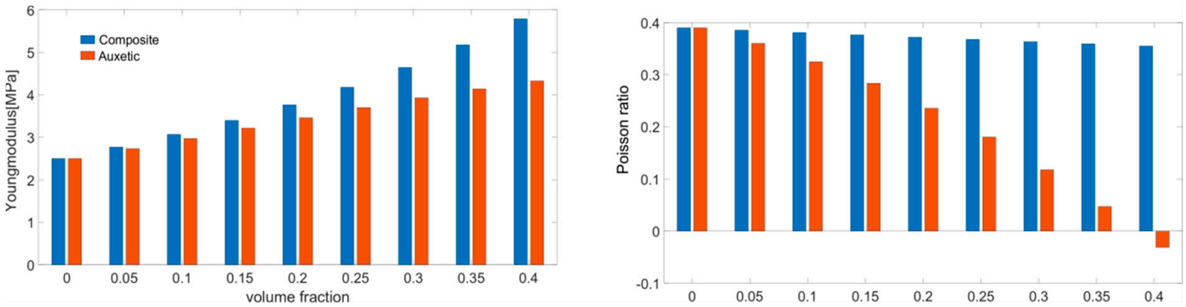

Figure Left - Young modulus and right - Poissons ratio of the materials as a function of volume fraction of inclusions.

Figure 4: Stress-strain function

The FEM simulations of tensile deformation for 5%, 10%, 20% and 30% were performed. The sample was virtually stretched to stretching ratio up to 30% elongation. The calculated parameter was stress–zz. The sample was uniaxially stretched in z-direction. The stress was measured at the plane with normal vector in z-direction. It is the equivalent of true stress measured by tensile test experiment.

Total stress (Cauchy stress) $_{zz}$ is calculated as the average stress of all elements. This value indicates the z-component of the normal stress that acts on the plane with the normal vector in the z-axis (normal stress). The dependence of the $_{zz}$ on the relative elongation of the whole sample in the z-axis is the tensile curve. This curve indicates the mechanical resistance of the specimens to tensile deformation.

The main text presents tensile curves for materials with 30% of inclusions. The tensile from simulation was compared to the tensile curve calculated from Mori Tanaka model.

Supplementary material presents all tensile curves for volume fractions from 0%, 5%, 10%, 20%, and 30% for reinforced composites and composites with auxetic inclusion.

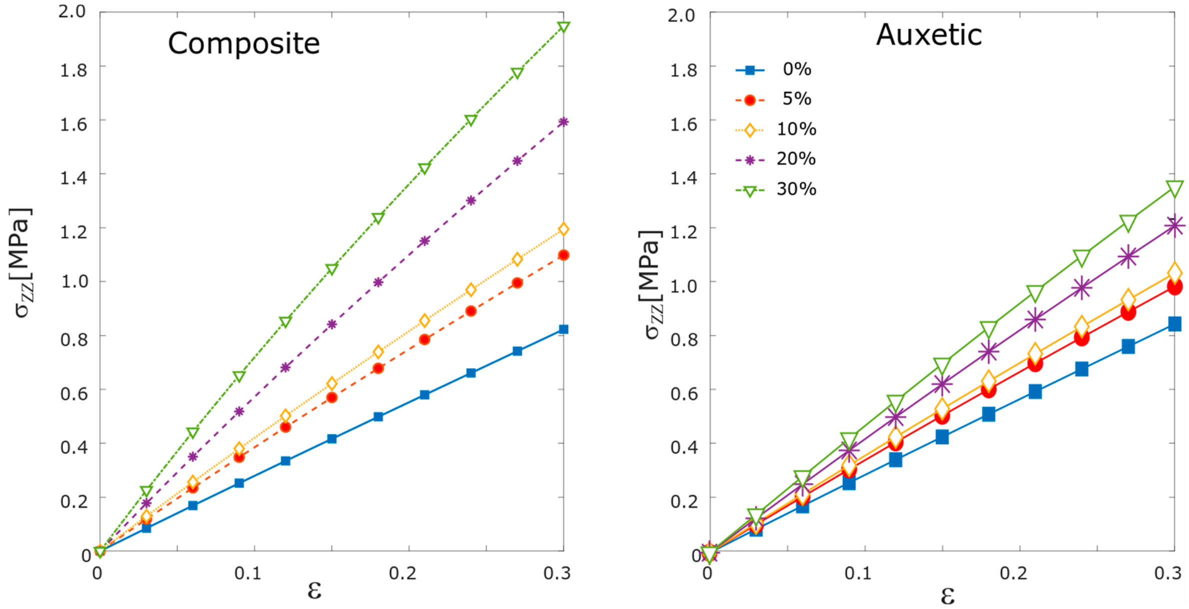

Figure True stress strain function of material filled with rigid particle (composite) and auxetic particle with various volume fraction.
