# Supplementary material for: Deformation of Gels with Spherical Auxetic Inclusions
Source: Gels. 2022 Oct 29;8(11):698. doi: 10.3390/gels8110698 (PMC9689618; doi:10.3390/gels8110698)
Supplement: Supplementary file 1 [file gels-08-00698-s001.zip › gels-1970954-supplementary/Zidek_et_al_Supplementary3.pdf]

Article: Deformation of the materials with spherical auxetic inclusions

Supplementary 3: Analogy to other metamaterials

Jan Zidek[a*], Petr Polacek, Josef Jancar[a]

[a]*CEITEC, Brno University of Technology, Purkynova 123, Brno, Czech Republic*

*corresponding author: jancar@fch.vutbr.cz

The space inside particle can be “invisible” for mechanical wave. The analogy of auxetic materials is an invisible cloak from metamaterials. It was theoretically described by Pendry [1]. Real invisibility cloak was proposed by Ni, Zhang et al [2].

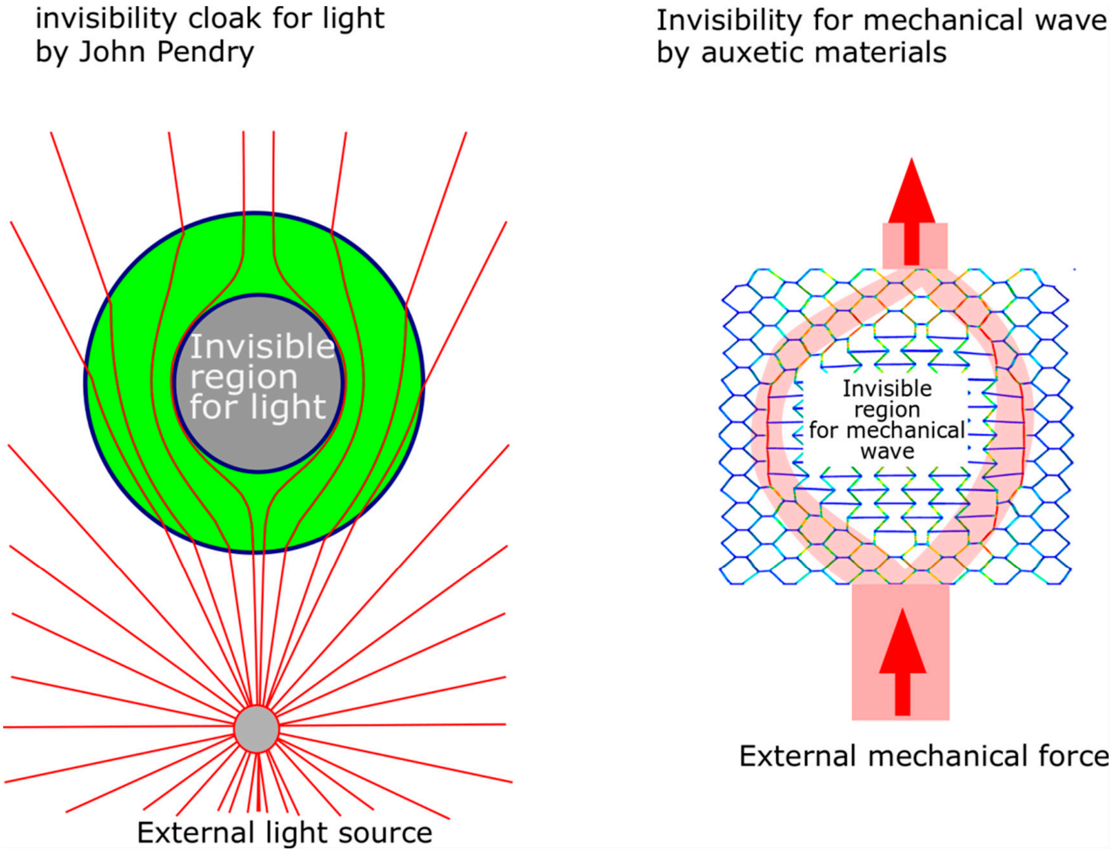

References

[1] Pendry, J. B., Schurig, D., Smith, D. R. (2006). Controlling Electromagnetic Fields. Science, 312(5781), 1780-1782. https://doi.org/10.1126/science.1125907.

[2] Ni, X., Wong, Z. J., Mrejen, M., Wang, Y., Zhang, X. (2015). An ultrathin invisibility skin cloak for visible light. Science, 349(6254), 1310-1314. https://doi.org/10.1126/science.aac9411.
